# Lineage Differentiation Potential of Different Sources of Mesenchymal Stem Cells for Osteoarthritis Knee

**DOI:** 10.3390/ph15040386

**Published:** 2022-03-22

**Authors:** Gollahalli Shivashankar Prajwal, Naveen Jeyaraman, Krishna Kanth V, Madhan Jeyaraman, Sathish Muthu, Sree Naga Sowndary Rajendran, Ramya Lakshmi Rajendran, Manish Khanna, Eun Jung Oh, Kang Young Choi, Ho Yun Chung, Byeong-Cheol Ahn, Prakash Gangadaran

**Affiliations:** 1 Research Fellow, Fellowship in Orthopaedic Rheumatology (FEIORA), Dr. Ram Manohar Lohiya National Law University, Lucknow 226010, Uttar Pradesh, India; prajwalgs1894@gmail.com (G.S.P.); naveenjeyaraman@yahoo.com (N.J.); 2 Indian Stem Cell Study Group (ISCSG) Association, Lucknow 110048, Uttar Pradesh, India; drsathishmuthu@gmail.com (S.M.); manishvenus@rediffmail.com (M.K.); 3 Department of Orthopaedics, Mallika Spine Centre, Guntur 522001, Andhra Pradesh, India; 4 Department of Orthopaedics, Atlas Hospitals, Tiruchirappalli 620002, Tamil Nadu, India; 5 Department of Orthopaedics, Government Medical College, Mahabubabad 506104, Telangana, India; krishna_kanth42@yahoo.com; 6 Department of Orthopaedics, Faculty of Medicine—Sri Lalithambigai Medical College and Hospital, Dr MGR Educational and Research Institute, Chennai 600095, Tamil Nadu, India; 7 Department of Biotechnology, School of Engineering and Technology, Sharda University, Greater Noida 201306, Uttar Pradesh, India; 8 Orthopaedic Research Group, Coimbatore 641001, Tamil Nadu, India; 9 Department of Medicine, Sri Venkateshwaraa Medical College Hospital and Research Centre, Puducherry 605102, Puducherry, India; sowndaryasreeraj8@gmail.com; 10 Department of Nuclear Medicine, School of Medicine, Kyungpook National University, Kyungpook National University Hospital, Daegu 41944, Korea; ramyag@knu.ac.kr; 11 Department of Orthopaedics, Government Medical College and Hospital, Dindigul 624001, Tamil Nadu, India; 12 Department of Orthopaedics, Prasad Institute of Medical Sciences, Lucknow 226010, Uttar Pradesh, India; 13 Department of Plastic and Reconstructive Surgery, CMRI, School of Medicine, Kyungpook National University, Kyungpook National University Hospital, Daegu 41944, Korea; fullrest74@hanmail.net (E.J.O.); kychoi@knu.ac.kr (K.Y.C.); hy-chung@knu.ac.kr (H.Y.C.); 14 BK21 FOUR KNU Convergence Educational Program of Biomedical Sciences for Creative Future Talents, Department of Biomedical Science, School of Medicine, Kyungpook National University, Daegu 41944, Korea

**Keywords:** mesenchymal stem cells, chondrogenesis, tissue engineering, cartilage, osteoarthritis

## Abstract

Tissue engineering and regenerative medicine (TERM) have paved a way for treating musculoskeletal diseases in a minimally invasive manner. The regenerative medicine cocktail involves the usage of mesenchymal stem/stromal cells (MSCs), either uncultured or culture-expanded cells along with growth factors, cytokines, exosomes, and secretomes to provide a better regenerative milieu in degenerative diseases. The successful regeneration of cartilage depends on the selection of the appropriate source of MSCs, the quality, quantity, and frequency of MSCs to be injected, and the selection of the patient at an appropriate stage of the disease. However, confirmation on the most favorable source of MSCs remains uncertain to clinicians. The lack of knowledge in the current cellular treatment is uncertain in terms of how beneficial MSCs are in the long-term or short-term (resolution of pain) and improved quality of life. Whether MSCs treatments have any superiority, exists due to sources of MSCs utilized in their potential to objectively regenerate the cartilage at the target area. Many questions on source and condition remain unanswered. Hence, in this review, we discuss the lineage differentiation potentials of various sources of MSCs used in the management of knee osteoarthritis and emphasize the role of tissue engineering in cartilage regeneration.

## 1. Introduction

Tissue engineering and regenerative medicine (TERM) have paved a way for treating musculoskeletal diseases in a less invasive manner by reducing the morbidity associated with the classical techniques and improving the functional quality of life of patients [1,2,3]. A regenerative medicine cocktail involves the usage of mesenchymal stem/stromal cells (MSCs), either uncultured or culture-expanded cells along with growth factors, cytokines, and exosomes and secretomes to provide better regeneration in degenerative diseases [4]. The tissue regenerated using these cocktails depends upon various factors, such as patient factors (age, sex, BMI, associated systemic disorders, ongoing medications), cellular factors (cell count, quality, and quantity of cells retrieved, cell separation technique), and component factors (amount of growth factors and cytokines released, lineage differentiation medium and its factors, scaffolds) [5,6].

The usage of MSCs has increased exponentially in musculoskeletal disorders in the areas of cartilage, tendon, nerve, and bony pathologies. Though cartilage is an avascular and aneural structure with an inferior intrinsic potential for regeneration, the augmentation, and appropriate induction of progenitor cells, along with necessary growth factors, help in cartilage regeneration [7,8,9,10]. The successful regeneration of cartilage depends on the selection of the appropriate sources (adipose tissue, bone marrow, placenta, amniotic fluid, peripheral blood, synovium, dental tissues, periosteum, endometrium, hematopoietic progenitors, and induced pluripotent cells), the quality, the quantity of MSCs, the frequency of MSCs to be injected, and the selection of the patient at an appropriate stage of the disease [11,12].

However, the optimal source of MSCs remains unproven. A serious gap in knowledge remains as to whether the currently used cellular treatments are beneficial in the long-term—apart from a short-term resolution of pain and improved quality of life—and whether any superiority exists in the sources of MSCs utilized in their potential to objectively regenerate the cartilage at the target area. Hence, in this review, we discuss the lineage differentiation potentials of various sources of MSCs used in the management of knee osteoarthritis and emphasize the role of tissue engineering in cartilage regeneration.

## 2. Differentiation Potential of MSCs

Various researchers have emphasized that MSCs enhance the regenerative microenvironment in diseased and degenerated tissues and organs. The regenerative potentials of MSCs are due to differentiation or transdifferentiation into parenchymal cells and the production of bio-active macromolecules at the site of action [13,14,15]. Several in vitro and in vivo studies indicated that MSCs promote collagen synthesis, induce neovasculogenesis, improve biomechanical strength, and reduce the scarring of regenerated tissues [16,17]. Clinically, the administered MSCs increase perfusion, decrease pain, contract, re-epithelise the wounds, and modulate inflammatory responses [14,16].

**Figure 1:**
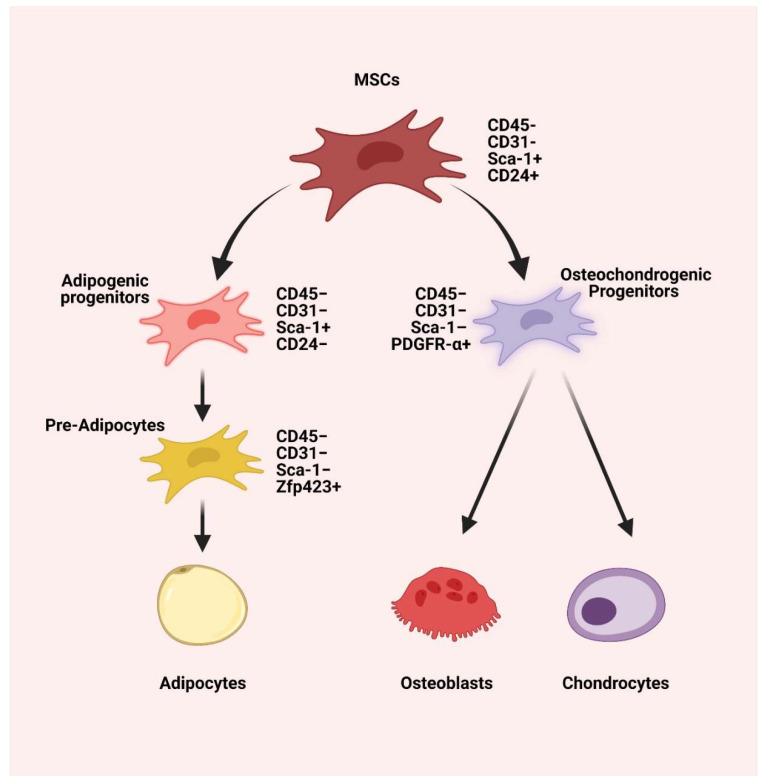
The trilineage differentiation potential of MSCs is characterized by CD45− CD31− Sca-1+ CD24+. The MSCs give rise to osteochondrogenic progenitors that are characterized by CD45− CD31− Sca-1– PDGFR-α+ and adipogenic progenitors characterized by CD45− CD31− Sca-1+ CD24−. The osteochondrogenic progenitors differentiate into chondrocytes and osteoblasts, while adipogenic progenitors differentiate into CD45− CD31− Sca-1− Zfp423+ pre-adipocytes, which leads to adipocytes differentiation [25]. Created with BioRender.com (accessed on 20 December 2021).

MSCs exhibit a hyper-regenerative potential (trilineage differentiation) and hypoimmunogenic potential (low MHC-1 and no MHC-2 expression) compared to other parenchymal cells, as shown in Figure 1 [18,19,20]. The regeneration potential of MSCs is much influenced by the donor’s age [21]. Various in vitro studies have proven the negative effects of aging on the regenerative potential of MSCs, such as (a) decreasing the number of cells during harvest or isolation, (b) decreasing the differentiative and proliferative potentials, (c) decreasing colony-forming units (CFUs), (d) decreasing MSC surface markers and miRNA markers expression, (e) decreasing immunomodulatory potential and (f) that it failed to express MSC morphology in cultures [22,23,24]. 

The keystone for adipogenic differentiation potential of MSCs is PPAR-γ. Adipogenic differentiation potential of MSCs occurs in two phases, namely (a) early phase (0–6 days) with the upregulation of CEBPB and D, SWI/SNF complex (BAF60b), SLUG, and FKHR factors, and (b) late phase (7–14 days) with the upregulation of CEBPB and D, mitogen-activated protein kinases, CDC2-associated protein, cyclin G1, PPAR-γ, CEBPA, FABP-α, and LPL [26]. The key factor responsible for chondrogenic differentiation is TGF-β1 [27]. Under suitable environments, MSCs transform to chondrogenic differentiation in two to three weeks with a plentiful extracellular matrix composed of type II collagen and aggrecan [28]. MSCs differentiation into the osteoblastic lineage from the common osteochondrogenic progenitor is orchestrated by the signaling from the osteocytes in the target milieu, even in the absence of the osteogenic media. The key markers of osteogenic differentiation are intracellular alkaline phosphatase and calcium deposition in the matrix [29]. In this review, we sum up the existing body of evidence on the lineage differentiation potential of the various sources of MSCs used for cartilage regeneration in osteoarthritis of the knee.

## 3. Bone Marrow-Derived MSCs (BM-MSCs)

Mohamed-Ahmed et al. exhibited the cellular yield, harvest, proliferation, and differentiation of BM-MSCs as negatively affected by the age of the donor [30]. BM-MSCs, having the higher expression of STRO-1, show a higher proliferation rate than Adipose tissue-derived MSCs (AD-MSCs) [30]. BM-MSCs exhibit early osteogenesis due to the formation of type 1 collagen, along with the higher expression of RUNX-2 and ALP activity on day 14 of the passage. In vitro studies stated that BM-MSCs possess a more increased osteogenic capacity than AD-MSCs due to the osteogenic gene expression and calcium deposition [30]. Due to an increased expression of aggrecan on day 28, BM-MSCs differentiate into the chondrocyte lineage more than AD-MSCs [31,32]. The cross-talk between BM-MSC-derived osteogenesis and adipogenesis is due to bone morphogenetic proteins(BMPs). BMP through BMPR-1A activates c/EBP-α and PPAR-γ via the Smad/p-38-MAPK pathways to differentiate MSC into adipocyte, whereas through BMPR-1B, it activates Runx-1, OSX, and PPAR-γ via the Smad/p-38-MAPK pathways to differentiate MSC into osteocyte. The mechanism of osteocyte differentiation of MSC by PPAR-γ is poorly understood [33]. PPAR-γ induction inhibits the β-catenin pathway during adipogenesis [34].

## 4. Adipose Tissue-Derived MSCs (AD-MSCs)

A study showed an elevated expression of CD34 and CD49d in AD-MSCs where CD34 expression is known to help in the prolonged cellular proliferation of MSCs [30]. AD-MSCs express Runx-1 and ALP activity after day fourteen on the passage. These expressions lead to a prolonged proliferation, maturation, and, finally, differentiation of AD-MSCs. The osteogenic differentiation of AD-MSCs is potentiated when AD-MSCs are subjected to mechanical stimulation along with osteogenic markers, such as vitamin D3, PDGF, and BMP-2 [35,36]. AD-MSCs are shown to activate adipogenesis through the induction of adiponectin, LPL, leptin, perilipin, and fatty acid-binding protein-1 by PPAR-γ and in addition, raised the lipid vesicle formation more than BM-MSCs [37]. Due to the reduced expression of TGF-β-R1, BMP-2, and BMP-4, the chondrogenic potential of AD-MSCs is decreased [38,39]. The chondrogenicity of AD-MSCs is characterized by the type 2 and 10 collagen, biglycan, aggrecan, and decorin genes expression in the differentiated cells [28]. AD-MSCs hold a potentially higher adipogenic differentiation than chondrogenic and/or osteogenic differentiation when compared with BM-MSCs [30,40,41]. 

## 5. Hematopoietic Stem Cells (HSCs)

Bone marrow contains MSCs and HSCs. HSCs are committed to hematopoietic lineages (erythropoiesis, leukopoiesis, and thrombopoiesis). HSCs are characterized by the presence of CD-45^+^, -34^+^, -31^+^, GATA-1^+^ and -3^+^, c-myb^+^, flk-1^+^/KDR^+^, and SCL^+^/TAL-1^+^ [42,43]. The homing effect of HSCs is maintained by stromal-derived factor -1 or the chemokine C-X-CR4 axis [44,45]. Upon the addition of specific lineage factors, HSCs differentiate into the particular lineage. HSC bound osteogenesis is mediated by BMP-2 and -6 through activation of PTH, Jagged-1 and -2, Delta-1 and -4, Hes-1 and -5, and Deltex ligand signaling [46,47,48]. Osteoblast trafficking in the HSC pool is maintained by osteopontin, angiopoietin-1, cysteine protease, cathepsin X, and C-X-CL-12 [49]. Chotinantakul et al. named osteoblasts and spindle-shaped N-cadherin+ osteoblastic cells as “Endosteal niche” [50]. The adipogenic potential of HSCs was poorly understood, yet the researchers have found that adipocyte is derived from monocyte/macrophage progenitor cells [51]. HSC-based adipogenic cells possess a Mac-1^low^ cell surface marker [52]. Gavin et al. explained the transition of hematopoietic lineage to adipogenic differentiation of HSCs by the integration of integrin-β1 [53]. There is no available literature on the role of HSC in chondrogenesis. 

## 6. Placental Derived MSCs (Pl-MSCs)

Though an immunologically temporary organ, the placenta being primitive and pluripotent, contains cellular components with stem cell-like activity and with higher potentiality for self-renewal and differentiation than other sources of MSCs [54,55,56,57]. Mesodermal (osteogenic, chondrogenic, and adipogenic) lineage differentiation has been demonstrated by human Wharton’s jelly (hWJ), decidua, and fetal membrane (FM)-derived MSC [57,58], whereas ectodermal (neurogenic) and endodermal (hepatogenic) lineages have been reported by FM-derived MSC and hWJ-MSC [59,60,61]. Pl-MSCs with CD-271^+^ differentiate into the osteogenic lineage [62]. Minimal oxygen tension inhibits Pl-MSC osteogenic differentiation. In addition, IGF-2 enhances differentiation through a relayed signaling cascade by IGF-1R/IR, PI3K, MEK1/2, and RUNX-2 phosphorylation more than IGF-1 [63]. Intraperitoneal injection of chorionic stem cells in a mouse model of osteogenesis imperfecta demonstrated a decreased number of fractures, as well as increased bone ductility and bone volume. Furthermore, the numbers of hypertrophic chondrocytes were increased and endochondral and intramembranous ossification-related endogenous genes were upregulated [64]. Increased secretion of glycosaminoglycans was observed when Pl-MSCs were seeded with the alginate/nCDHA/RGD mixed gel, which provides a 3D construct in the form of engineered cartilage tissue [65]. The TGF-β1-immobilized human fibroblast-derived extracellular matrix (ECM) with heparin provides a microenvironment for chondrogenic differentiation of Pl-MSCs in 3D collagen spheroid [66]. Chondrocyte ECM enriches the chondrogenesis of Pl-MSCs and is further enriched by preculture with chondrocyte-derived ECM [67]. 

## 7. Amniotic Fluid-Derived MSCs (Af-MSCs)

Af-MSC populations are a heterogeneous mixture with differentiated and undifferentiated progenitor cells derived from the fetus [68,69]. Af-MSCs are culture expandable and express CD-29, -44, -73, -90, -105, and SSEA4 with over 90% of the cells being positive for OCT-4 [68]. They express embryonic stems cell markers, such as TRA-1-60, TRA-1-81, SSEA3, and SSEA [70]. These fetal-derived cells retained their multi-differentiation capacities (adipogenic, chondrogenic, and osteogenic). They show a higher differentiation potential compared to adult stem cells [70]. Af-MSCs show similar characteristics with primordial germ cells expressing Sox17,c-Kit, STELLA, FGF-8, Nanos, DAZL, VASA, FRAGILIS, SSEA1, and Pum-2 [71]. Cloned lines of CD-117 selected Af-MSCs to modulate immune responses in chondrogenesis [72]. Compared to BM-MSCs, Af-MSCs cells generated less cartilaginous matrix after three weeks of TGF-β1 supplementation in pellet and alginate-based culture and hence, Af-MSCs have the ability to differentiate along the chondrogenic lineage [73]. Human Af-MSCs act as an important source for the induction of chitosan-based chondrogenesis [74]. Activation of calcium-sensing receptors by calcimimetic R-568 induces the osteogenic differentiation of Af-MSCs [75]. Wnt signaling acts as a key regulator in an osteogenic lineage of Af-MSCs by the upregulation of disheveled-2 expression, and the adipogenic lineage of Af-MSCs by the downregulation of disheveled-2 expression [76]. SOX-2 and ID-2 are the key targets of Nanog and POUSF-1, which are involved in the ossification and adipogenesis of Af-MSCs [77]. The exosome, miR-26a mediates the adipogenic lineage of Af-MSCs via PTEN, CyclinE1, and CDK6 [78]. 

## 8. Peripheral Blood-Derived MSCs (PB-MSCs)

PB-MSCs are obtained by mobilizing BM-MSCs to peripheral blood by giving G-CSF, which is called “blood mobilization” [79,80,81]. PB-MSCs constitute a heterogeneous population of cells containing MSCs, HSCs, immature blasts, and progenitor cells [82,83]. PB-MSCs possess CD-146 and 104b expression when compared with BM-MSCs [84]. The MSC count in PB-MSCs remains low when compared with other sources of MSCs. Though a higher cellular count prevails with BM-MSC, with 2 mL of peripheral blood, it is estimated that approximately 5 million cells PB-MSCs can be expanded in vitro for reparative procedures [85]. PB-MSCs express RUN-2, osterix, osteopontin, osteonectin, and *COLIA1* during osteoblastic differentiation [86]. PB-MSCs upregulate the chondrogenic genes associated with the chondrogenic differentiation of MSCs present in the infrapatellar fat pad, increase the number of MSCs, cause native chondrocyte migration, and accelerate the rate of cellular movement [87]. Lyahyai et al. [88] and Spaas et al. [89] demonstrated that BM-MSCs possess a higher differentiation potential for osteogenic and chondrogenic lineages than PB-MSCs. Chong et al. reported that PB-MSCs possess higher adipogenic differentiation than BM-MSCs and similar chondrogenic differentiation than BM-MSCs [81]. In a rat model, while comparing with BM-MSCs, PB-MSCs possess a greater chondrogenic differentiation ability, whereas BM-MSCs possess greater osteogenic, adipogenic, and proliferative ability [90]. PB-MSCs seeded with hydroxyapatite polylactic-glycolic acid induce osteogenesis at a 4 mm calvarial bone defect in a rat model, which was evaluated by micro-CT [91].

## 9. Synovium-Derived MSCs (Sy-MSCs)

The minimally explored source of MSC in cellular therapy remains the synovium-derived MSCs. Literature reported that the synovium lining (the outer layer contains type A macrophage-like synoviocytes) of the knee joint provides an excellent source of Sy-MSCs [92,93,94,95]. These type A cells stain positive for CD-68 & -14, and collagen III, V & VI [94]. Due to limited senescence, Sy-MSCs have to be expanded in monolayer culture in vitro. Sy-MSCs possess superior chondrogenicity due to increased expression of CD-44, SOX-9, COMP, aggrecan, and collagen 1, 10, and 11 [94,96,97]. The cross-talks between ERK1/2 and SOX-9 stimulate the chondrogenic differentiation of Sy-MSCs [98,99,100]. In pellet culture media, Sy-MSCs regenerate an increased number of cartilage pellets when matched with BM-MSCs. A study reported that under in vitro conditions, the chondrogenic capability of Sy-MSCs was greater than that of periosteum-derived MSCs [101]. In six OA patients, Mizuno et al. observed a greater proliferation and chondrogenesis in the MSCs present in the perivascular region of the synovium, whereas poorer chondrogenesis was observed in the MSCs from the stromal part of the synovium [102]. In a rabbit model, Bami et al. demonstrated osteogenesis, chondrogenesis, myogenesis, and ethnogenesis with Sy-MSCs [103]. Fibrous synovium contains more MSCs than adipose synovium. Though retarded potential for adipogenesis, Katagiri et al. demonstrated adipogenesis of Sy-MSCs with the synovial tissue harvested during total knee arthroplasty [104]. 

## 10. Dental Tissue-Derived MSCs (D-MSCs)

Stem cells of dental origin (dental pulp, periodontal ligament, human exfoliated deciduous teeth, apical papilla, dental follicle, and gingiva) form a good therapeutic concept in regenerating tissues, cartilage, and bones. In addition to specific growth factors, ECM proteins, and transcriptional factors, dental pulp-derived MSCs (DP-MSCs) differentiate into multilineages, namely adipogenesis, osteogenesis, chondrogenesis, neurogenesis, and dentinogenesis [105,106]. D-MSCs possess immunophenotypes, such as CD-44, -73, -90, -105, -271, and STRO-1 like BM-MSCs, AD-MSCs, and Sy-MSCs [107,108,109]. Scaffold-assisted chondrogenesis by D-MSCs increases the procollagen type 2 and 10, alkaline phosphatase, aggrecan, and SOX-9 genes; in addition, decreases the Nanog, Slug, Twist, and Snail genes [110,111]. Distal-less homeobox 5 (DLX5) and C8 (HOXC8) boosted the chondrogenic differentiation of stem cells of the apical papilla (SCAPs). DLX5 and HOXC8 overexpression lead to upregulation of transcriptional activity of COL2, COL5, and SOX-9, which induces chondrogenesis [112]. The BMP-4/Smad signaling cascade is necessary for the osteogenic differentiation of DP-MSCs. This may be inhibited by tumor necrosis factor-inducible protein-6 (TSG-6) [113]. Amir et al. demonstrated a significant increase in DP-MSCs metabolism in 2 weeks of culture when added with chitosan, which is responsible for proliferation and early osteogenic differentiation of DP-MSCs [114]. Various studies demonstrated that DP-MSCs have regenerative potential to differentiate into functional osteoblasts in vitro and were able to produce extracellular matrix components [115,116]. Laino et al. demonstrated the differentiation of DP-MSCs into osteoblast precursors to living autologous fibrous bone (LAB) tissue [117]. Once the LAB tissue was transplanted, they were able to give rise to adult bone cells in immunocompromised rats [118,119]. 

## 11. Periosteum-Derived MSCs (P-MSCs)

The periosteum, an outer covering of bone, contains a cambium layer which is composed of mesenchymal progenitor cells, which are called periosteum-derived MSCs. P-MSCs hold prolonged proliferation and differentiation capacities, and a retention of differentiation ability in the in vitro culture condition as well as the in vivo condition [120,121]. P-MSCs from load-bearing sites have more osteogenic capability than flat bones [122]. After the fracture, the quiescent P-MSCs induce chondrogenesis and osteogenesis. In addition, they help in long-term integration together with native bone [123,124]. An analysis of the lineage of P-MSCs demonstrated that P-MSCs from the Prx-1 positive mesenchymal lineage add to cartilage and bone within the callus [125]. CD-90^+^ P-MSCs showed greater osteogenic potency than unsorted P-MSCs, either in vitro or in vivo [126]. Therefore, CD-90^+^ P-MSCs could be an ideal cell source with greater osteogenic potency for bone regeneration. Periosteal progenitors differentiate into chondrocytes in the presence of TGF-β3 along with atelocollagen, as evaluated by type 2 collagen staining [127]. TGF-β1 and IGF-1 improve in vitro cartilage regeneration, subperiosteal administration of TGF-β1 and IGF-1 in aged rabbits, the phenotypic stability, and cellular count in the cambium layer of periosteum [128].

## 12. Endometrium-Derived MSCs (En-MSCs)

En-MSCs are readily available in reproductive women 12 times a year with greater proliferation ability [129]. En-MSCs are a heterogeneous population of the cellular mixture as that of AD-MSCs [130,131,132]. The stromal cell activity markers of En-MSCs are CD-146^+^/PDGF-Rβ^+^ and SUD-2 [133]. Chan et al. reported 0.22% of endometrial epithelial and 1.25% of endometrial stromal cells demonstrate clonogenicity, proving the presence of progenitor cells in the human endometrium [132]. A cellular population from a menstrual fluid containing CD-146^+^ PDGFR-β^+^ constitutes En-MSCs (functionalis and basalis of human endometrium) [132,133,134]. En-MSCs retain embryonic stem cell markers up to 20 cycles of subculturing and maintain a normal karyotype after 12 passages of subculture [135]. However, with the greater proliferative capacity of Me-MSCs due to the higher expression of Oct-4, menstrual fluid-derived MSCs (Me-MSCs) possess inferior mineralization of cells in osteogenic medium fortified with fetal bovine serum when compared with BM-MSCs. To improve osteogenesis, Me-MSCs are combined with platelet release to produce the desired action [136]. Me-MSCs exhibit enhanced chondrogenesis when admixed with TGF-β3, BMP-2, and activin. Though En-MSCs differentiate into trilineage components, the ability of chondrogenic potential was lower when compared to BM-MSCs in an equine model [137]. When En-MSCs pellets were cultured along with dexamethasone and TGF-β2 or TGF-β3 for 3 to 21 days, these cells proliferate and resemble chondrocytes with increased expression of sulfated glycosaminoglycans and type 2 collagen [138]. 

## 13. Induced Pluripotent Stem Cells (iPSCs)

iPSCs are engineered and reprogrammed pluripotent stem cells of adult somatic cell origin, which can retain the properties of embryonic stem cells (ESCs). The most challenging task in iPSCs is the time-effective generation of a significant amount of functional MSCs. iPSCs possess similar phenotypic characteristics to ESCs. iPSC markers are 5T4, ABCG2, Activin RIB, ALP, B18R, E-Cadherin, Cbx2, CD9, CD30/TNFRSF8, CD-117/c-kit, CDX2, CHD1, Cripto, DNMT3B, DPPA-2, -4, & -5, EpCAM/TROP1, ERR-β, ESGP, F-box protein 15, FGF-4 & -5, FoxD3, GBX2, GCNF, GDF-3, Integrin-α6, -α6β1, -α6β4, & -β1, KLF-4 & -5, L1TD1, Lefty-1 & -A, LIN-28A, -28B, & -41, c-Maf, c-Myc, Nanog, Oct-3/4, -4A, & -4B, Podocalyxin, Rex-1, Smad 2/3, SOX-2, SSEA-1, -3, & -4, STAT-3, Stella, SUZ12, TBX-2, -3, & -5, TEX19, TEX19.1, THAP11, TRA-1-60(R), TRA-1-81, TROP-2, UTF1, VISTA/B7-H5/PD-1H, and ZIC-3 [139]. Kang et al. compared differentiation lineages and stemness’ of two iPSC lines (mRNA-iPSC-MSC-YL001 and lenti-iPSC-MSC-A001) and BM-MSCs [140]. iPSC lineages (avg 40%) exhibited higher proliferation rates than BM-MSCs (avg 27%), similar surface marker gene expression, and lower colony-forming capability in soft agar, suggesting lower tumorigenic capabilities [141]. iPSCs exhibited adequate osteogenic and chondrogenic properties and were less efficient in adipogenicity when compared to BM-MSCs [140]. iPSC-derived MSCs on hydroxyapatite-coated polymer scaffolds induce the osteoclastic differentiation of iPSC-macrophage (by *NFATC1*, *CATK*, *CTR*, and *TRAP5b*) and possess stronger osteogenic activity of human iPSCs compared to low HA or PLLA/PLGA alone [142]. Bioglass induces the stimulation of osteogenesis of iPSCs in vitro, which was assayed by ALP levels and real-time PCR [143]. The osteogenic differentiation of iPSCs from human gingival fibroblasts was notably increased when admixed with nanohydroxyapatite/chitosan/gelatine 3D scaffolds [144]. Prolonged pulses in the low-frequency electromagnetic field on iPSCs induce osteogenesis under in vitro conditions [145]. Engineered chondrogenesis from iPSCs exhibits the same marginal expression of chondrocytes hypertrophic markers (*PTH1R*, *COL10A1*, *IBSP,* and *ALPL*) like cartilage from articular cartilage. Collagen X was hardly detectable in the iPSC-cartilage. Furthermore, this was thirty-fold lower than in hypertrophic cartilage derived from MSCs [146]. Chondrocytes derived from iPSC-MSCs exhibited improved histology and expressed less IL-1*β*, TNF-*α*, and MMP13 than control cartilage [147]. iPSCs exhibit a similar ability of adipogenesis when compared with embryonic stem cells and express the transcription of C/EBP-α, PPARγ2, leptin, and aP2 markers [148]. 

## 14. Comparison of Lineage Differentiation of Various Sources of MSCs

Upon comparing the regenerative differentiation potential of the various sources of MSCs used in osteoarthritis knee, it is evident that the more conventional sources commonly used nowadays, such as BM-MSCs, stand only next to the most potential P-MSCs, as shown in Table 1. Although sources, such as Af-MSCs, PB-MSCs, D-MSCs, P-MSCs, and iPSCs are also comparable to the BM-MSCs, they have certain limitations. Af-MSCs, being an allogenic source, have the demerit of possible mismatch in their usage to the recipient. PB-MSCs, although appear more appealing for clinical use, it needs prior administration of the mobilization regimen, and the number of cells obtained from the harvest is always lower than the collection from the native marrow from where the mobilization occurs. The utilization of D-MSCs requires prior collection and storage of deciduous teeth. Although sources such as Sy-MSCs and P-MSCs appear promising, their harvest requires invasive procedures and subsequent culturing before administration to attain an appropriate quantity of cells before administration, thereby preventing their use in a single surgical session. 

## 15. Challenges in the Source of MSC Identification

**Figure 2:**
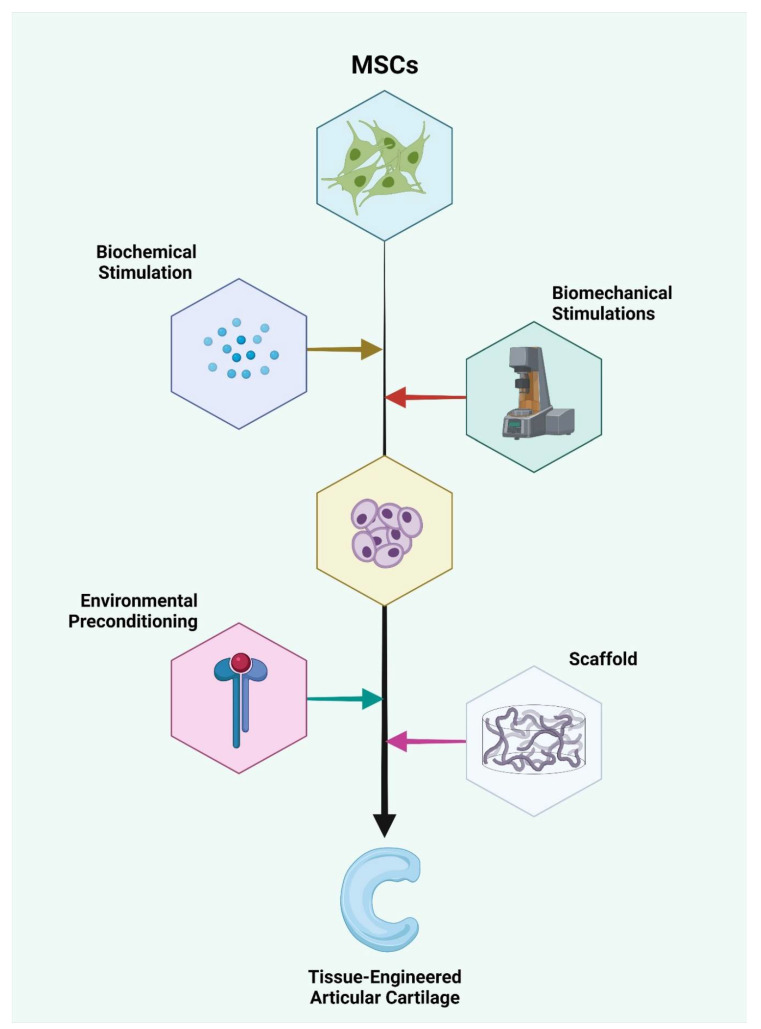
Lineage manipulation of different sources of MSCs with biochemical stimulation using mediators, such as IGF, FGF, TGF-β, BMP, Loxl2, c-ABCs, and biomechanical stimulations, such as compressive, tensile, or shear loading, along with the necessary hydrostatic pressure results in the formation of the chondrocyte tissue complex. The chondrocyte complex is further subjected to environmental preconditioning using chemokines, such as IL-1, to sensitize them to the target milieu when delivered with or without supporting scaffold, resulting in the formation of the tissue-engineered articular cartilage. Created with BioRender.com (accessed on 20 December 2021).

One must be reminded that the source of the MSCs used in the above-mentioned studies are prepared to meet the harsh in vivo environments using certain selected agents to sensitize and adapt them to the aggressive pathological milieu they are about to encounter. For example, in cases with secondary osteoarthritis of rheumatoid origin, the MSCs used for therapy should be exposed to strong inflammatory cytokines, such as IL-1. Similarly, preconditioning of the MSCs to a similar inflammatory environment during culture will optimize their behavior to the pathological milieu of administration. These methods tailor the treatment methods appropriate to the patient’s needs, thereby paving the way for a “personalized medicine” that has a high rate of optimal results upon usage. Lineage manipulation of different sources of MSCs with biochemical stimulation using mediators, such as IGF, FGF, TGF-β, BMP, Loxl2, c-ABCs, and biomechanical stimulations, such as compressive, tensile, or shear loading, along with the necessary hydrostatic pressure, results in the formation of the chondrocyte tissue complex. The chondrocyte complex is further subjected to environmental preconditioning using chemokines, such as IL-1, to sensitize them to the target milieu when delivered with or without supporting scaffolding, resulting in the formation of tissue-engineered articular cartilage, as shown in Figure 2. Hence, before the administration of any source of MSCs with multilineage potential, manipulation of their culture environment with appropriate chemical mediators would not only tune them, but would also guide them towards differentiation to the appropriate lineage of choice at the target site. 

## 16. Conclusions

We have discussed the lineage differentiation potential of various sources of MSCs that stand as an eligible contender for use in osteoarthritis of the knee and comparatively evaluated their trilineage differentiation potential. Although sources such as P-MSCs appear more promising, the BM-MSCs stand to be more practical for utilization in clinical scenarios. Having discussed the benefits of all the available sources of MSCs, we recommend future research on their comparative differentiation potential in the pathological milieu tailored to the patient’s conditions to obtain optimal results upon their usage. 

## Figures and Tables

**Table 1:** Comparison of lineage differentiations of various sources of MSCs.

Source of MSCs	Osteogenesis	Chondrogenesis	Adipogenesis
BM-MSC	++++	+++	+
AD-MSC	++	++	++++
HSC	+	+/−	+
Pl-MSC	+++	++	++
Af-MSC	++	+++	++
PB-MSC	++	+++	++
Sy-MSC	+++	++++	+
D-MSC	++++	+++	+
P-MSC	++++	+++	+
En-MSC	++	++	++
iPSC	+++	+++	+++

BM-MSC—bone marrow-derived MSC; AD-MSC—adipose tissue-derived MSC; HSC—hematopoietic stem cells; Pl-MSC—placental derived MSC; Af-MSC—amniotic fluid-derived MSC; PB-MSC—peripheral blood-derived MSC; Sy-MSC—synovium-derived MSC; D-MSC—dental tissue-derived MSC; P-MSC—periosteum-derived MSC; En-MSC—menstrual fluid-derived MSC; iPSC—induced pluripotent stem cells. +: low potential; ++: moderate potential; +++: high potential; ++++: very high potential; −: no potential.

## Data Availability

Data sharing not applicable.
